# The predictive value of prognostic nutritional index on early complications after robot-assisted radical cystectomy

**DOI:** 10.3389/fsurg.2022.985292

**Published:** 2022-11-16

**Authors:** Yiduo Wang, Xun Lu, Yue Gao, Ning Liu, Hua Jiang, Shuqiu Chen, Ming Chen

**Affiliations:** ^1^ Department of Urology, Affiliated Zhongda Hospital of Southeast University, Nanjing, China; ^2^ Surgical Research Center, Institute of Urology, School of Medicine, Southeast University, Nanjing, China

**Keywords:** prognostic nutritional index, robot-assisted radical cystectomy (RARC), complications, predictive value, early

## Abstract

**Objective:**

The purpose of the study was to evaluate the predictive value of prognostic nutritional index (PNI) on early complications (within 30-day) after robot-assisted radical cystectomy (RARC) and urinary diversion.

**Patients and methods:**

Patients underwent RARC and urinary diversion between November 2018 and December 2021 in our centre were screened in this retrospective study. Baseline characteristics and perioperative data were recorded. Early complications after surgery were classified according to Clavien-Dindo system. Univariate and multivariate logistic analysis were performed to decide the potential factors associated with post-RARC complications. The receiver operating characteristic (ROC) curve was conducted to determine the predictive value of PNI on early overall and major complications after RARC.

**Results:**

Overall 139 men and 13 women with a median age of 69 years and mean BMI of 24.4 kg/m^2^ were included in this study. As for urinary diversion, most patients (*n* = 111, 73%) received cutaneous ureterostomy, 36 patients (23.7%) underwent orthotopic neobladder and 5 patients (3.3%) received ileal conduit. The incidence of postoperative complication rate was 44.7%, which included 82.2% minor complications and 17.8% major complications. Further univariate and multivariate logistic analyses demonstrated that hypertension (OR = 2.96, 95% CI: 1.24–7.07, *P* = 0.015), PNI (OR = 0.73, 95% CI: 0.62–0.86, *P* < 0.001), and CCI (OR = 1.44, 95% CI: 1.01–2.06, *P* = 0.047) were independent risk factors of early complications after RARC. Moreover, PNI (OR = 0.72, 95% CI: 0.60–0.86, *P* < 0.001) was also the predictor of major complications after RARC. The ROC curve demonstrated that PNI (AUC = 0.829; AUC = 0.840) has a great predictive value in early overall and major complications after RARC.

**Conclusion:**

PNI can be an early alert for RARC patients thus aiding in closer monitoring and postoperative management.

## Introduction

Bladder cancer is the 10th most common malignant tumor with both high morbidity and mortality around the world (1). Owing to its high progressive, radical cystectomy (RC) and urinary diversion is the recommended curative therapy in muscle invasive bladder cancer and high risk non-muscle invasive bladder cancer (2). While the advances in surgical techniques, RC and urinary diversion remains to be one of the most complicated surgery in urology.

Previous studies have reported that compared to open radical cystectomy (ORC), robot-assisted radical cystectomy (RARC) has decreased overall postoperative complication rate and achieved comparable oncologic outcomes (3–5). Moreover, RARC is also significantly associated with lower perioperative transfusion rate, fewer major complication rate and shorter length of stay (LOS) than ORC (6–10). To reduce and prevent the occurrence of postoperative complications after RARC, it is vital to determine potential factors associated with postoperative complications.

Many cancer patients are accompanied by malnutrition status, which leads to high postoperative complications, adverse survival outcomes and high economic burden (11). Thus, it is important to evaluate the preoperative nutrition status before surgery. The prognostic nutritional index (PNI) is widely used in many cancers to predict postoperative outcomes by integrating serum albumin level and lymphocyte count, which is routinely measured before surgery (12–14). Yu et al. reported that patients with lower PNI was associated with a higher rate of postoperative pulmonary complications after RC in a retrospective study (15). Meanwhile, our previous study has also found that PNI is the independent risk factors associated with early urinary tract infection after RC (16). However, few studies have evaluated preoperative nutrition status and postoperative outcomes in RARC.

Therefore, we reported the overall and major postoperative complication (within 30-day) rate after RARC, and identified factors associated with early postoperative complications after RARC. Moreover, the predictive value of PNI on postoperative outcomes was also measured.

## Patients and methods

### Study design

Patients who diagnosed with bladder cancer and underwent RARC between November 2018 and December 2021 were included in this retrospective study. The inclusion criteria included that age over 18 years; pathologically confirmed urothelial carcinoma; and no history of other malignant tumors and immune system diseases. Patients lack of complete data or those who underwent RARC combined with other surgery at the same time were also excluded. This study was approved by the local institutional review board and informed consent was waived due to its retrospective nature.

### Treatment

All patients included in this study underwent RARC and standard pelvic lymph node dissection (PLND) at our tertiary institution. For patients with impaired preoperative renal function or tumor invasion of adjacent organs, cutaneous ureterostomy (CU) or ileal conduit (IC) were performed. While orthotopic neobladder (ONB) was reconstructed decided by patients and their treating urologists.

### Data collection

We screened our hospital database system and collected baseline characteristics of patients, including gender, age, Charlson comorbidities Index (CCI), American Society of Anesthesiologists Physical Status (ASA), body mass index (BMI), preoperative hydronephrosis, neoadjuvant chemotherapy, comorbidities (smoking status, hypertension, and diabetes), history of abdominal surgery, history of intravesical instillation, and preoperative laboratory tests (hemoglobin, albumin, and lymphocyte). Data of operation time, estimated blood loss (EBL), numbers of dissected lymph node, intraoperative transfusion rate and LOS were also reviewed. Oncologic outcomes included pathological tumor stage, lymphovascular invasion and surgical margin. Tumor stage was standardized according to the 8th American Joint Committee on Cancer (AJCC) tumor, lymph node, metastasis (TNM) system (17).

### Definitions and outcomes

PNI is the index to assess the nutritional status and reflect the immune function of patients, which is calculated by 10× serum albumin (g/dl) + 0.005 × total lymphocyte count (/mm^3^). Overall complications within 30-day after surgery were defined as early complications and were classified according to Clavien-Dindo system. The definition of minor complications were grade II or less, while grade III or greater were major complications (18).

The primary outcome was early complication rate after RARC. The secondary outcome was risk factors associated with early complications.

### Statistical analysis

For continuous variables, they were shown as mean ± standard deviation (SD) or median ± interquartile range (IQR) according to the data distribution. The *t*-test or the Mann–Whitney *U* test were conducted as appropriate. For categorical variables, they were represented as frequencies or percentages and were compared by chi-squared or Fisher's exact tests. The univariate analyses was performed to select potential risk factors associated with early complications. If the variables were calculated as *P* < 0.10 in univariate analysis, then they were further entered into the multivariate logistic analyses. The results were shown as odds ratios (OR) and 95% confidence intervals (95% CI).

The best cut-off value of PNI was determined by the receiver operating characteristic (ROC) curve, then the whole cohort was divided into low PNI and high PNI group according to the optimal cut-off value. In multivariate logistic analysis, three models were constructed to further assess the relationship between PNI and overall and major complications. Basic model: adjusted for age, gender, BMI, hydronephrosis, hypertension, diabetes and smoking; core model: basic model variables plus urinary diversion type, history of abdominal surgery, operation time, estimated blood loss and numbers of dissected lymph node; extended model: core model variables plus AJCC stage, T stage, N stage, history of intravesical instillation and neoadjuvant chemotherapy.

The predictive ability of PNI was evaluated by ROC curve. The results were shown as the area under the curve (AUC). The ROC curve was constructed by MedCalc program (version 20.015). Finally, *P* < 0.05 was considered as statistically significant. All the statistical analysis was performed by the software Stata (version 15.1).

## Results

### Baseline characteristics and perioperative data

**Figure 1:**
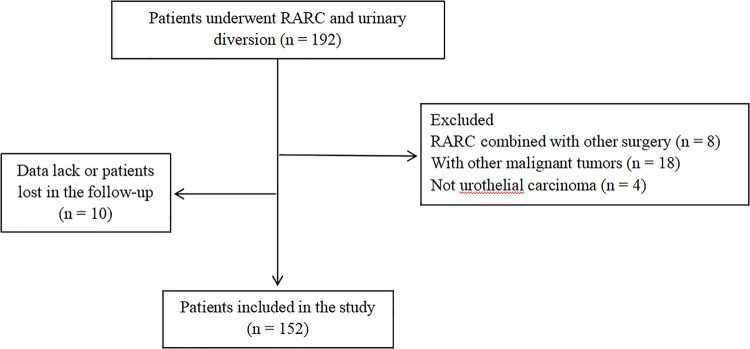
The flow chart of protocol in the study.

All patients underwent RARC were screened, and 152 patients were finally included in the study according to the inclusion and exclusion criteria. The flow chart was shown in Figure 1.

A total of 139 men and 13 women with a median age of 69 years (IQR, 63–75) and mean BMI of 24.4 kg/m^2^ were included. The mean values of preoperative laboratory tests of hemoglobin and albumin were 131.1 ± 22.7, and 37.7 ± 4.5. The comorbidities of smoking, hypertension, and diabetes were present in 12.5%, 42.8%, and 15.8% of patients, respectively. Eleven patients (7.2%) received neoadjuvant chemotherapy preoperatively and 41 patients (27.0%) had previous history of abdominal surgery. Patients’ physical condition is not very well in general, as 17 patients (11.2%) had ASA > 2 and 48 patients (31.6%) had CCI ≥ 4. Among patients underwent RARC, 43 patients (28.2%) received intravesical instillation previously. About pathological results, most of patients (69.7%) were localized tumor, and only 16.4% of patients were lymph node positive, and few patients present lymphovascular invasion. For urinary diversion, 111 patients (73.0%) received CU, while 5 patients (3.3%) received IC, and 36 patients (23.7%) underwent ONB, respectively. Besides, median operation time (OT) was 307.5 min (IQR, 275–383.8) and median EBL was 100 ml (IQR, 100–200). Intraoperative transfusion was recorded in 15 patients (9.9%). The median LOS after operation was 17 days (IQR, 12–25). The detailed baseline characteristics and perioperative data were showed in Table 1.

**Table 1:** Baseline characteristics and perioperative data of included patients.

Variable	Result
Total, *n*	152
Age, years, median (IQR)	69 (63–75)
Gender, *n* (%)	
Male	139 (91.5%)
Female	13 (8.5%)
Preoperative hemoglobin, g/L, mean ± SD	131.1 ± 22.7
Albumin, g/L, mean ± SD	37.7 ± 4.5
BMI, kg/m^2^, mean ± SD	24.4 ± 3.2
PNI, mean ± SD	45.6 ± 6.4
Hypertension	
Absence	87 (57.2%)
Presence	65 (42.8%)
Diabetes	
Absence	128 (84.2%)
Presence	24 (15.8%)
Smoking status	
Absence	133 (87.5%)
Presence	19 (12.5%)
Neoadjuvant chemotherapy	
Absence	141 (92.8%)
Presence	11 (7.2%)
ASA score, *n* (%)	
1–2	135 (88.8%)
3–4	17 (11.2%)
Charlson comorbidity index, *n* (%)	
0–1	21 (13.8%)
2–3	83 (54.6%)
4–5	43 (28.3%)
>5	5 (3.3%)
History of abdominal surgery, *n* (%)	
Absence	111 (73.0%)
Presence	41 (27.0%)
Preoperative hydronephrosis, *n* (%)	
Absence	115 (75.7%)
Presence	37 (24.3%)
T stage	
1–2	106 (69.7%)
3–4	46 (30.3%)
N stage	
N−	127 (83.6%)
N+	25 (16.4%)
Lymphovascular invasion	
Absence	99 (65.1%)
Presence	53 (34.9%)
Urinary diversion, *n* (%)	
Cutaneous ureterostomy	111 (73.0%)
Ileal conduit	5 (3.3%)
Orthotopic neobladder	36 (23.7%)
History of intravesical instillation	
Absence	109 (71.7%)
Presence	43 (28.2%)
Intraoperative transfusion	
Absence	137 (90.1%)
Presence	15 (9.9%)
Estimated blood loss, ml, median (IQR)	100 (100–200)
Operation time, minutes, median (IQR)	307.5 (275–383.8)
Dissected lymph node, median (IQR)	14 (10–20)
Postoperative hospital stay, days, median (IQR)	17 (12–25)

### Data of complications

The incidence of overall complications within 30-day was 44.7%. Totally, 84 complications were recorded in 68 patients, including 15 cases (17.9%) of Grade I complication, 54 cases (64.3%) of Grade II complication, 11 cases (13.1%) of Grade III complication, and 4 cases (4.7%) of Grade IV complication, respectively. No complications greater than Grade IV were found. Generally, complications were classified as minor (Grade I or Grade II) or major (Grade III or greater) type according to Clavien-Dindo system. Based on the severity of complications, 54 patients (79.4%) were recorded with minor complications and 14 patients (20.6%) were recorded with major complications. The calculated overall major complication rate was 9.2%. The detailed data of postoperative complications were listed in Table 2.

**Table 2:** Postoperative complications following RARC.

Variable	Result
Patients with complications, *n* (%)	68 (44.7%)
Minor complications, Clavien-Dindo grade I–II	54 (79.4%)
Major complications, Clavien-Dindo grade III–V	14 (20.6%)
Total number of complications, *n* (%)	84
Grade I	15 (17.9%)
Grade II	54 (64.3%)
Grade III	11 (13.1%)
Grade IV	4 (4.7%)
Complications, *n* (%)	
Infectious complications	24 (28.5%)
Gastrointestinal complications	17 (20.2%)
Transfusion	14 (16.7%)
Cardiovascular complications	7 (8.3%)
Lymphatic leakage	5 (6.0%)
Anastomosis site stricture	3 (3.6%)
Deep venous thrombosis	2 (2.4%)
Others	12 (14.3%)

**Figure 2:**
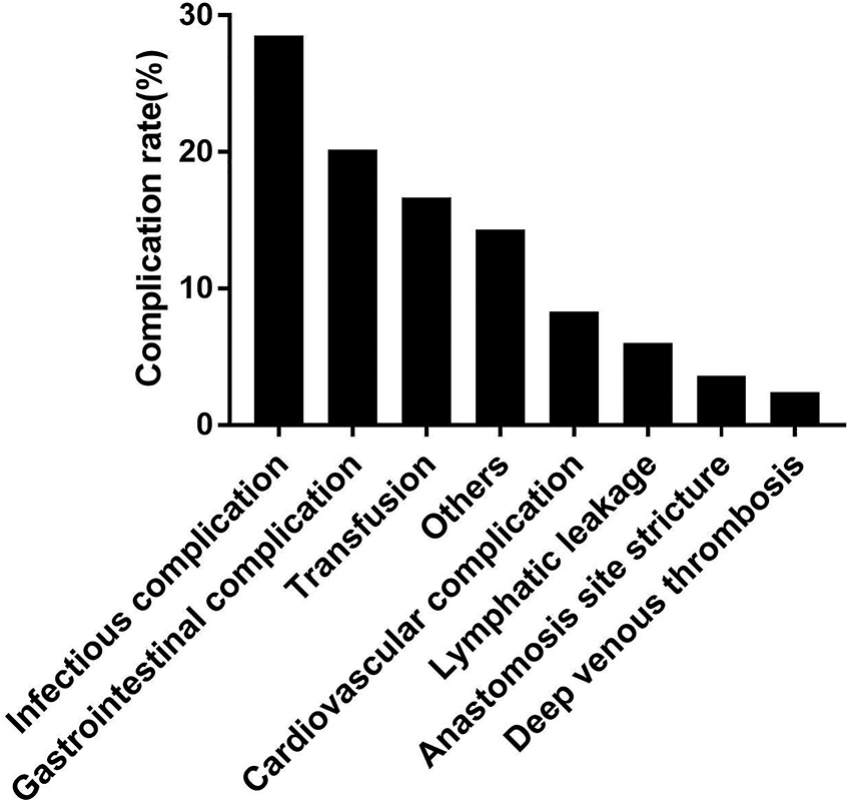
Type of early complications after RARC and urinary diversion.

Among these complications, the most frequent complications were infectious complications (28.5%), followed by gastrointestinal complications (20.2%), transfusion (16.7%), cardiovascular complications (8.3%), lymphatic leakage (6.0%), anastomosis site stricture (3.6%), and deep venous thrombosis (2.4%). The detailed type of complications was shown in Figure 2. Moreover, more major and gastrointestinal complications happened in IC and ONB group.

### Risk factors associated with complications

**Figure 3:**
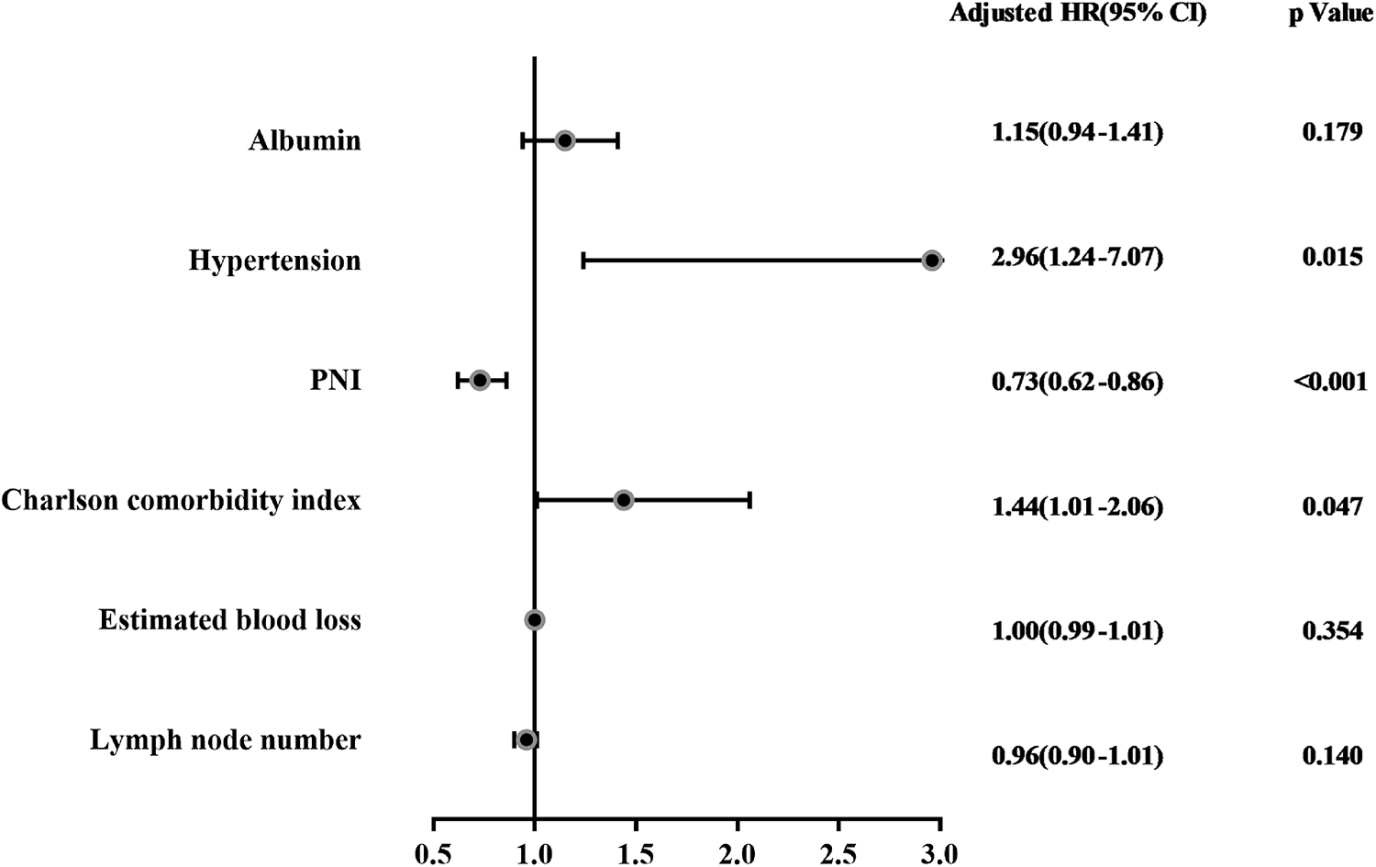
The forest plot of multivariate analysis.

Univariate analysis was conducted to select the factors associated with postoperative complications. The results showed that preoperative albumin level (OR = 0.77, 95% CI: 0.70–0.85, *P* < 0.001), hypertension (OR = 2.39, 95% CI: 1.24–4.61, *P* = 0.010), PNI (OR = 0.78, 95% CI: 0.72–0.85, *P* < 0.001), CCI (OR = 2.02, 95% CI: 1.49–2.75, *P* < 0.001), estimated blood loss (OR = 1.00, 95% CI: 1.00–1.01, *P* = 0.097), and dissected lymph node numbers (OR = 0.94, 95% CI: 0.90–0.98, *P* = 0.009) were associated with postoperative complications (Table 3). Then, these identified factors were further entered into multivariate analysis. Multivariate analysis results showed that hypertension (OR = 2.96, 95% CI: 1.24–7.07, *P* = 0.015), PNI (OR = 0.73, 95% CI: 0.62–0.86, *P* < 0.001), and CCI (OR = 1.44, 95% CI: 1.01–2.06, *P* = 0.047) were independent risk factors of early complications (Table 4). The forest plot of multivariate analysis was shown in Figure 3. The univariate and multivariate analysis results were also shown in Supplementary Tables S1, S2. The results indicated that hypertension (OR = 5.06, 95% CI: 1.20–21.39, *P* = 0.028) and PNI (OR = 0.70, 95% CI: 0.58–0.85, *P* < 0.001) were also predictors of major complications after RARC.

**Table 3:** Univariate analysis of overall postoperative complications after RARC.

Variable	OR	95% CI	*P*
Gender			
Female	1.00	-	-
Male	0.67	0.21–2.10	0.492
Albumin	0.77	0.70–0.85	**<0** **.** **001**
Hypertension			
Absence	1.00	-	-
Presence	2.39	1.24–4.61	**0** **.** **010**
Diabetes			
Absence	1.00	-	-
Presence	0.70	0.29–1.72	0.439
BMI	0.95	0.86–1.05	0.318
History of smoking			
Absence	1.00	-	-
Presence	0.69	0.26–1.86	0.461
Preoperative hemoglobin	1.00	0.98–1.01	0.658
PNI	0.78	0.72–0.85	**<0** **.** **001**
Charlson comorbidity index	2.02	1.49–2.75	**<0** **.** **001**
ASA	1.01	0.61–1.69	0.961
Urinary diversion			
Cutaneous ureterostomy	1.00	-	-
Ileal conduit	0.81	0.13–5.06	0.825
Neobladder	0.98	0.46–2.08	0.950
Preoperative hydronephrosis			
Absence	1.00	-	-
Presence	0.92	0.44–1.95	0.834
History of abdominal surgery			
Absence	1.00	-	-
Presence	0.95	0.46–1.96	0.900
Operation time	1.00	1.00–1.01	0.508
Estimated blood loss	1.00	1.00–1.01	0.097
Lymph node number	0.94	0.90–0.98	**0** **.** **009**
History of intravesical instillation			
Absence	1.00	-	-
Presence	0.97	0.48–1.97	0.932
Neoadjuvant chemotherapy			
Absence	1.00	-	-
Presence	1.53	0.45–5.24	0.500

Bold values indicate that the variable is significant (*P* < 0.05).

**Table 4:** Multivariate analysis of overall postoperative complications after RARC.

Variable	OR	95% CI	*P*
Albumin	1.15	0.94–1.41	0.179
Hypertension	2.96	1.24–7.07	**0** **.** **015**
PNI	0.73	0.62–0.86	**<0** **.** **001**
Charlson comorbidity index	1.44	1.01–2.06	**0** **.** **047**
Estimated blood loss	1.00	0.99–1.01	0.354
Lymph node number	0.96	0.90–1.01	0.140
Type of diversion (IC and ONB vs. CU)	1.97	0.83–4.71	0.126

Bold values indicate that the variable is significant (*P* < 0.05).

To further validate the significance of PNI in overall and major complications, three models were constructed as basic model, core model and extended model. As indicated in Supplementary Tables S3, S4, PNI was consistently a predictor factor for overall and major complications, whether in the basic model (aOR = 0.29, 95% CI: 0.13–0.61, *P* = 0.001; aOR = 0.07, 95% CI: 0.01–0.41, *P* = 0.003), core model (aOR = 0.29, 95% CI: 0.13–0.64, *P* = 0.002; aOR = 0.07, 95% CI: 0.01–0.41, *P* = 0.003) or extended model (aOR = 0.25, 95% CI: 0.11–0.58, *P* = 0.001; aOR = 0.06, 95% CI: 0.01–0.54, *P* = 0.011).

### Predictive value of PNI on early complications

**Figure 4:**
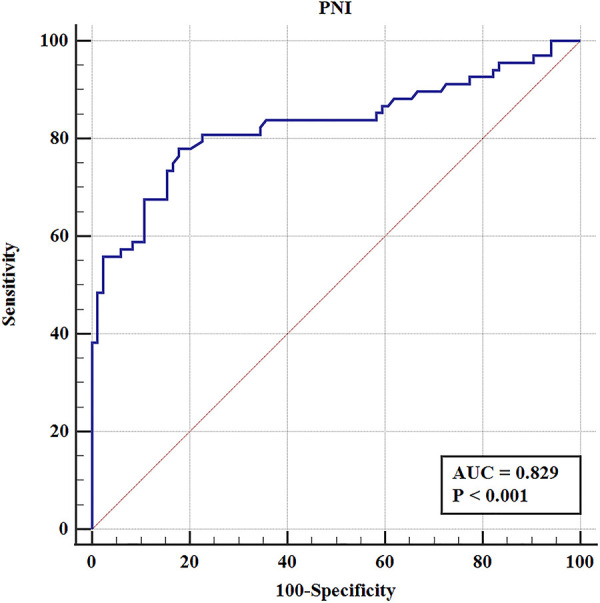
The ROC curve of PNI in overall RARC complications.

**Figure 5:**
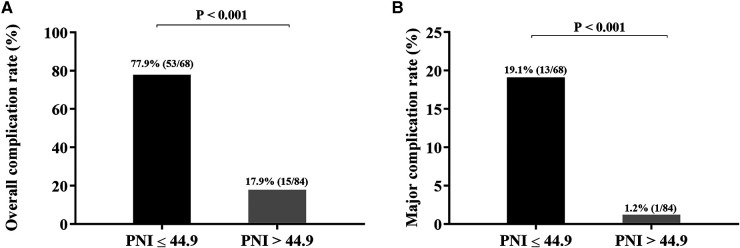
(A) Overall complication rate (B) Major complication rate between low and high PNI group.

To elucidate the effect of PNI on complications after RARC, the ROC curve was constructed. The results showed that the AUC, sensitivity and specificity were 0.829%, 77.9%, and 82.1%, respectively (Figure 4). According to the best Youden index, the optimal cut-off of PNI was 44.9, and then we divided the entire cohort into low PNI (PNI ≤ 44.9) group and high PNI (PNI > 44.9) group. Of the 152 patients, 68 patients (44.7%) had PNI ≤ 44.9 and 84 patients (55.3%) had PNI > 44.9. As shown in Figure 5, the incidence of overall and major complications in low PNI group were significantly higher than high PNI group (77.9% vs. 17.9%, *P* < 0.001; 19.1% vs. 1.2%, *P* < 0.001). Moreover, the constructed ROC curve also demonstrated that PNI was a great predictor of major RARC complications (Supplementary Figure S1). The AUC, sensitivity and specificity were 0.840%, 66.7%, and 86.9%, respectively.

In comparison with high PNI group, patients in low PNI group were older (71.5 vs. 67 years, *P* < 0.001), and had longer LOS after surgery (19.5 vs. 15 days, *P* = 0.025). However, there were no significant difference in gender, pathological stage, intraoperative transfusion rate, dissected lymph node numbers, estimated blood loss and operation time (Table 5). The ROC curve of age and PNI were compared to further confirm the predictive value of these variables. Statistical significance of AUC was calculated by Delong's test. As shown in Supplementary Figure S2, the results showed that the predictive value of PNI was better than age in overall and major complications (*P* < 0.001 and *P* = 0.006).

**Table 5:** Association of PNI with pathological stage and perioperative outcome.

Variable	Low PNI group	High PNI group	*P*
	PNI ≤ 44.9	PNI > 44.9	
Total, *n* (%)	68 (44.7%)	84 (55.3%)	
Gender			0.249
Female	8 (11.8%)	5 (6.0%)	
Male	60 (88.2%)	79 (94.0%)	
Age, years, median (IQR)	71.5 (67–79)	67 (60–72)	**<0** **.** **001**
pT			0.390
0–2	45 (66.2%)	61 (72.6%)	
3–4	23 (33.8%)	23 (27.4%)	
pN			0.215
N−	54 (79.4%)	73 (86.9%)	
N+	14 (20.6%)	11 (13.1%)	
Hypertension			0.335
Absence	36 (52.9%)	51 (60.7%)	
Presence	32 (47.1%)	33 (39.3%)	
Diabetes			0.437
Absence	59 (86.8%)	69 (82.1%)	
Presence	9 (13.2%)	15 (17.9%)	
LVI			0.921
Absence	44 (64.7%)	55 (65.5%)	
Presence	24 (35.3%)	29 (34.5%)	
Urinary diversion			0.883
Cutaneous ureterostomy	51 (75.0%)	60 (71.4%)	
Ileal conduit	2 (2.9%)	3 (3.6%)	
Neobladder	15 (22.1%)	21 (25%)	
Intraoperative transfusion			0.210
Absence	59 (86.8%)	78 (92.9%)	
Presence	9 (13.2%)	6 (7.1%)	
Major complications			**<0** **.** **001**
Absence	55 (80.9%)	83 (98.8%)	
Presence	13 (19.1%)	1 (1.2%)	
LNN, median (IQR)	12 (9–17.8)	15 (10–20.8)	0.055
EBL, ml, median (IQR)	100 (100–237.5)	100 (100–200)	0.299
OT, minutes, median (IQR)	300 (261.3–360)	312.5 (280–390)	0.324
LOS, days, median (IQR)	19.5 (13.3–29.8)	15 (11–22)	**0** **.** **025**

Bold values indicate that the variable is significant (*P* < 0.05).

## Discussion

In present study, we reported that the incidence of early overall complications in patients undergoing RARC was 44.7% and hypertension, PNI, and CCI were independent risk factors of postoperative complications. Moreover, the impact of PNI on complications after RARC was also evaluated. The optimal cut-off point of PNI for predicting complications after RARC was 44.9. The incidence of complications was significant higher in low PNI (PNI ≤ 44.9) group than high PNI (PNI > 44.9) group. Furthermore, low PNI was associated with older age, high major complications rate and longer LOS.

The percentage of RARC usage has constantly increased since its introduction. A recent multi-institutional retrospective study showed that the trend of RARC usage increased exponentially in the European centers, which increased from 2% in 2006–2008 to 50% in 2015–2018. While in the North American centers, the trend remained stable between 70% and 80% vary 2006–2018 (6). Oncologic outcomes and postoperative complications rate are critical issue for RARC. Previous series demonstrated that RARC was non-inferior to ORC in oncologic outcomes. However, with regard to early complications rate (within 30-day), the reported results vary from 30% to 78%. A retrospective study by Pruthi et al. reported that the overall complications rate was 36% and major complication rate was 8% among consecutive 100 patients and most patients underwent ileal conduit diversion (19). Likewise, according to Hayn et al., the early complication rate was 40% and there were 13% high grade complications in a prospective study. Meanwhile, they also found that gastrointestinal, infectious, and genitourinary complications were most common among these patients (20). In contrast, Lau et al. showed that the overall complication rate was 78% and high grade complication rate was 35% (21). The potential explanation for the difference of complication rate vary centers may be the different characteristics, urinary diversion types, and postoperative management protocols among patients. Till now, the study with the highest volume of RARC patients was conducted by Soria et al. (7). The multicenter retrospective cohort study included 1,197 patients with bladder cancer underwent RARC at 10 academic centers between 2000 and 2017. The cumulative early overall complication rate was 42% within 30-day after surgery. Similar results were obtained in our study, which showed that early overall and major complication rate were 44.7% and 9.2%, respectively. With respect to the complication type, we also reported consistent results as previous studies. The most common complications in our study were infectious, gastrointestinal complications and postoperative transfusion.

Many studies have explored factors of early complications after RARC. Mermier et al. (22) identified that anticoagulant therapy and ureteroenteric anastomosis-type Wallace II were associated with a higher rate of overall complications after RARC. While opioid-free analgesia and intracorporeal diversion were protective factors of overall complications. Nazmy et al. (23) found that higher ASA score, lower preoperative hematocrit and continent urinary diversion type were associated with higher complication rate. Lee et al. (24) proposed that previous intravesical instillation, preoperative hemoglobin and estimated blood loss were predictors of RARC complications. However, controversy remains between the urinary diversion type and overall postoperative complications. According to Abe et al. (25), there was no significant difference in the overall complication rate between ileal conduit and neobladder (72% vs. 74%, *P* = 0.591). Moreover, Lenfant et al. (26) compared complications of extracorporeal and intracorporeal urinary diversion in patients with bladder cancer who underwent RARC in a multi-institutional retrospective study. The results indicated that overall complication rate, operation time, LOS, positive surgical margin, and dissected lymph node numbers did not significantly differ among the two cohorts. Larger analyses are needed to confirm this issue. According to other literature on complications after RARC, age, neoadjuvant chemotherapy, transfusion, ASA score and CCI were common predictors of any grade complications.

Notably, we identified PNI as a predictive factor for early complications after RARC along with hypertension and CCI. PNI was calculated by total serum albumin level added 5 fold lymphocyte count, which was firstly introduced by Onodera et al. (27) when assessing 189 gastrointestinal surgical patients those who were malnourished and treated by total parenteral nutrition preoperatively. The results showed that this index provided an accurate, quantitative estimate of operative risk. When PNI was below 40, patients had high risk to die within the next 2 months. It is common that most malignant tumor patients were malnourished, which might lead to adverse postoperative outcomes. Numerous studies have confirmed that patients with low PNI level was associated with high postoperative complication rate, such as colorectal cancer (13), esophageal cancer (11) and intrahepatic cholangiocarcinoma (28). The mechanisms for decreasing in serum albumin concentrations are reduction in synthesis, increased catabolism and renal or gut losses (29). Albumin maintains colloid osmotic pressure, regulates substance binding and transport, affects coagulation, inhibits thrombosis, and accelerates repairing and healing of damaged tissues (30). Many pathologies such as cancer, sepsis, and surgery could lead to lymphopenia, which reflects the immunosuppression state. Persistent lymphopenia is also associated with increased mortality in surgical patients (31). To sum up, the index of PNI before RARC could aid in accurate risk stratification and tailoring of care schedule in order to potentially improving postoperative outcomes.

There are several limitations of this study. First, this study was a single center retrospective analysis, which inevitably lead to selection bias. Second, the value of the study was confined by its relatively small quantity of data. Third, while we showed the results of complications over time, more accurate results should be analyzed of learning curve of the surgeon. Finally, owing to few major complications occurred in our cohort, no predictors were analyzed for major complications, which might be more important. Nevertheless, our study is meaningful as it not only reemphasizes existing predictors of complications after RARC, but also presents a novel nutrition index of PNI, which has a high predictive ability. However, more well-designed prospective and multicenter studies are needed to reconfirm our results.

## Conclusion

In summary, hypertension, CCI, and PNI were independent risk factors of postoperative complications after RARC. PNI had a great predictive value in postoperative complications which could aid in closer monitoring and postoperative management.

## Data Availability

The raw data supporting the conclusions of this article will be made available by the authors, without undue reservation.
