# Supplementary material for: The predictive value of prognostic nutritional index on early complications after robot-assisted radical cystectomy
Source: Front Surg. 2022 Nov 16;9:985292. doi: 10.3389/fsurg.2022.985292 (PMC9708885; doi:10.3389/fsurg.2022.985292)
Supplement: Supplementary file 1 [file Table1.docx]

Supplementary table 1 Univariate analysis of major postoperative complications after RARC

| Variable | OR | 95%CI | P |
| --- | --- | --- | --- |
| Gender |  |  |  |
| Female | 1.00 | - | - |
| Male | 0.31 | 0.08-1.30 | 0.111 |
| Albumin | 0.94 | 0.83-1.06 | 0.302 |
| Hypertension |  |  |  |
| Absence | 1.00 | - | - |
| Presence | 2.98 | 0.97-9.20 | **0.057** |
| Diabetes |  |  |  |
| Absence | 1.00 | - | - |
| Presence | 0.80 | 0.17-3.82 | 0.784 |
| BMI | 0.98 | 0.82-1.16 | 0.784 |
| History of smoking |  |  |  |
| Absence | 1.00 | - | - |
| Presence | 1.00 | - | - |
| Preoperative hemoglobin | 0.99 | 0.97-1.01 | 0.359 |
| PNI | 0.76 | 0.67-0.87 | **< 0.001** |
| Charlson comorbidity index | 1.57 | 1.03-2.38 | **0.036** |
| ASA | 1.32 | 0.57-3.06 | 0.522 |
| Urinary diversion |  |  |  |
| Cutaneous ureterostomy | 1.00 | - | - |
| Ileal conduit | 2.53 | 0.26-24.82 | 0.427 |
| Neobladder | 1.26 | 0.37-4.30 | 0.709 |
| Preoperative hydronephrosis |  |  |  |
| Absence | 1.00 | - | - |
| Presence | 1.15 | 0.34-3.84 | 0.825 |
| History of abdominal surgery |  |  |  |
| Absence | 1.00 | - | - |
| Presence | 0.98 | 0.29-3.28 | 0.977 |
| Operation time | 1.00 | 0.99-1.01 | 0.414 |
| Estimated blood loss | 1.00 | 1.00-1.01 | **0.033** |
| Lymph node number | 0.95 | 0.87-1.02 | 0.171 |
| History of intravesical instillation |  |  |  |
| Absence | 1.00 | - | - |
| Presence | 3.33 | 1.13-9.85 | **0.030** |
| Neoadjuvant chemotherapy |  |  |  |
| Absence | 1.00 | - | - |
| Presence | 0.91 | 0.11-7.62 | 0.928 |
