# Supplementary material for: The predictive value of prognostic nutritional index on early complications after robot-assisted radical cystectomy
Source: Front Surg. 2022 Nov 16;9:985292. doi: 10.3389/fsurg.2022.985292 (PMC9708885; doi:10.3389/fsurg.2022.985292)
Supplement: Supplementary file 2 [file Table2.docx]

Supplementary table 2 Multivariate analysis of major postoperative complications after RARC

| Variable | OR | 95%CI | P |
| --- | --- | --- | --- |
| History of intravesical instillation | 4.01 | 0.98-16.44 | 0.054 |
| Hypertension | 5.06 | 1.20-21.39 | **0.028** |
| PNI | 0.70 | 0.58-0.85 | **< 0.001** |
| Charlson comorbidity index | 1.01 | 0.54-1.89 | 0.967 |
| Estimated blood loss | 1.00 | 0.99-1.01 | 0.186 |
| Type of diversion(IC and ONB vs. CU) | 3.78 | 0.76-18.80 | 0.104 |
