# Supplementary material for: The predictive value of prognostic nutritional index on early complications after robot-assisted radical cystectomy
Source: Front Surg. 2022 Nov 16;9:985292. doi: 10.3389/fsurg.2022.985292 (PMC9708885; doi:10.3389/fsurg.2022.985292)
Supplement: Supplementary file 3 [file Table3.docx]

Supplementary table 3 Adjusted odds ratio of overall complications after RARC was calculated according to PNI

|  | Basic model | Core model | Extended model |
| --- | --- | --- | --- |
| PNI | aOR (95% CI) P | aOR (95% CI) P | aOR (95% CI) P |
| Low | 1 - | 1 - | 1 - |
| High | 0.29 (0.13-0.61) 0.001 | 0.29 (0.13-0.64) 0.002 | 0.25 (0.11-0.58) 0.001 |

Adjusted covariates: Basic model: adjusted for age, gender, BMI, hydronephrosis, hypertension, diabetes and smoking; core model: basic model variables plus urinary diversion type, history of abdominal surgery, operation time, estimated blood loss and numbers of dissected lymph node; extended model: core model variables plus AJCC stage, T stage, N stage, history of intravesical instillation and neoadjuvant chemotherapy.
