# Supplementary material for: The predictive value of prognostic nutritional index on early complications after robot-assisted radical cystectomy
Source: Front Surg. 2022 Nov 16;9:985292. doi: 10.3389/fsurg.2022.985292 (PMC9708885; doi:10.3389/fsurg.2022.985292)
Supplement: Supplementary file 5 [file Table5.docx]

Supplementary table 5 Postoperative complications segregated by the type of diversion

|  | CU (n=111) | IC (n=5) | ONB (n=36) |
| --- | --- | --- | --- |
| Patients with complications, n (%) | 50 | 2 | 16 |
| Minor complications | 41 (82.0) | 1 (50.0) | 12 (75.0) |
| Major complications | 9 (18.0) | 1 (50.0) | 4 (25.0) |
| Total number of complications, n (%) | 53 | 2 | 29 |
| Infectious complications | 16 (30.2) | 1 (50.0) | 7 (24.1) |
| Gastrointestinal complications | 8 (15.1) | 0 (0) | 9 (31.0) |
| Transfusion | 12 (22.6) | 0 (0) | 2 (6.9) |
| Cardiovascular complications | 5 (9.4) | 0 (0) | 2 (6.9) |
| Lymphatic leakage | 4 (7.5) | 0 (0) | 1 (3.4) |
| Anastomosis site stricture | 1 (1.9) | 0 (0) | 2 (6.9) |
| Deep venous thrombosis | 2 (3.8) | 0 (0) | 0 (0) |
| Others | 5 (9.4) | 1 (50.0) | 6 (20.7) |
