# Supplementary figures and images for: The predictive value of prognostic nutritional index on early complications after robot-assisted radical cystectomy
Source: Front Surg. 2022 Nov 16;9:985292. doi: 10.3389/fsurg.2022.985292 (PMC9708885; doi:10.3389/fsurg.2022.985292)

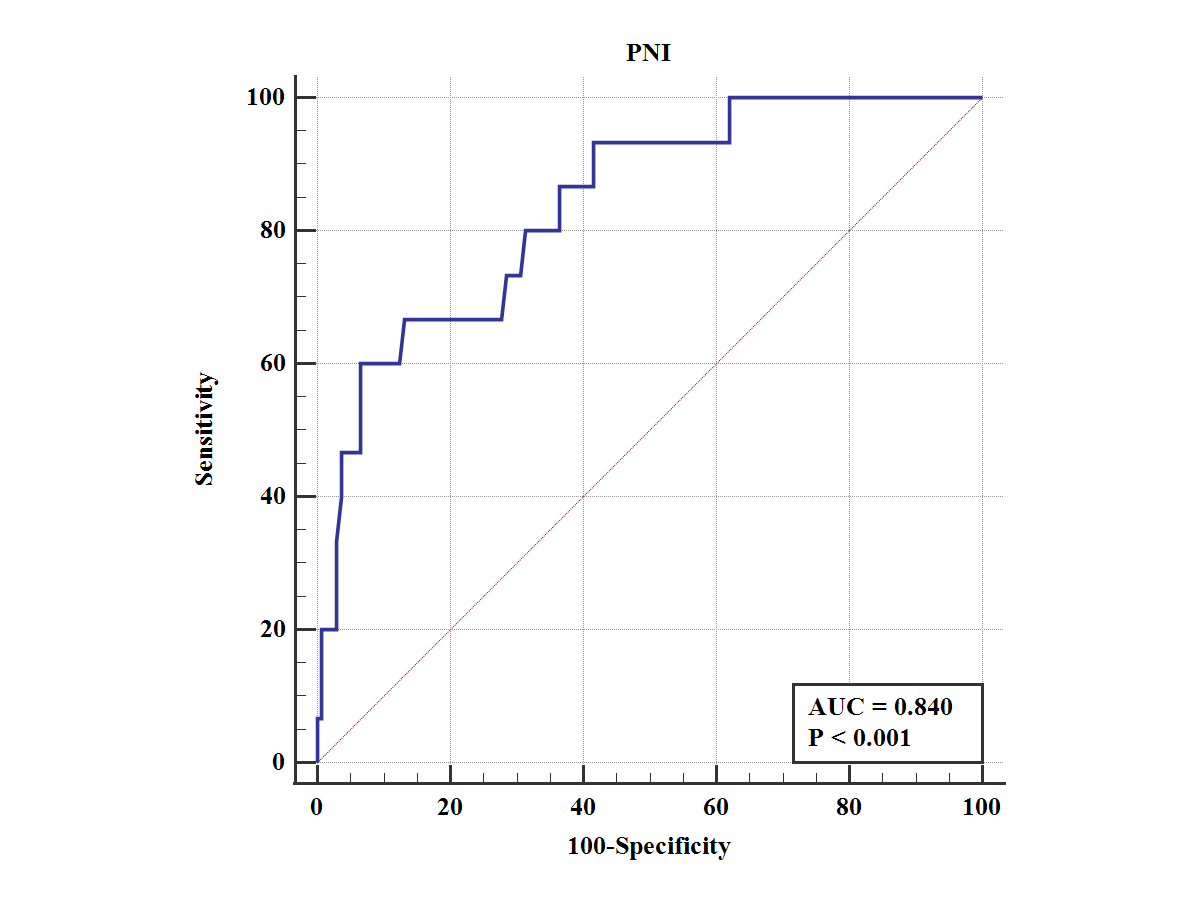

Supplement: Supplementary file 6 [file Image1.tif]

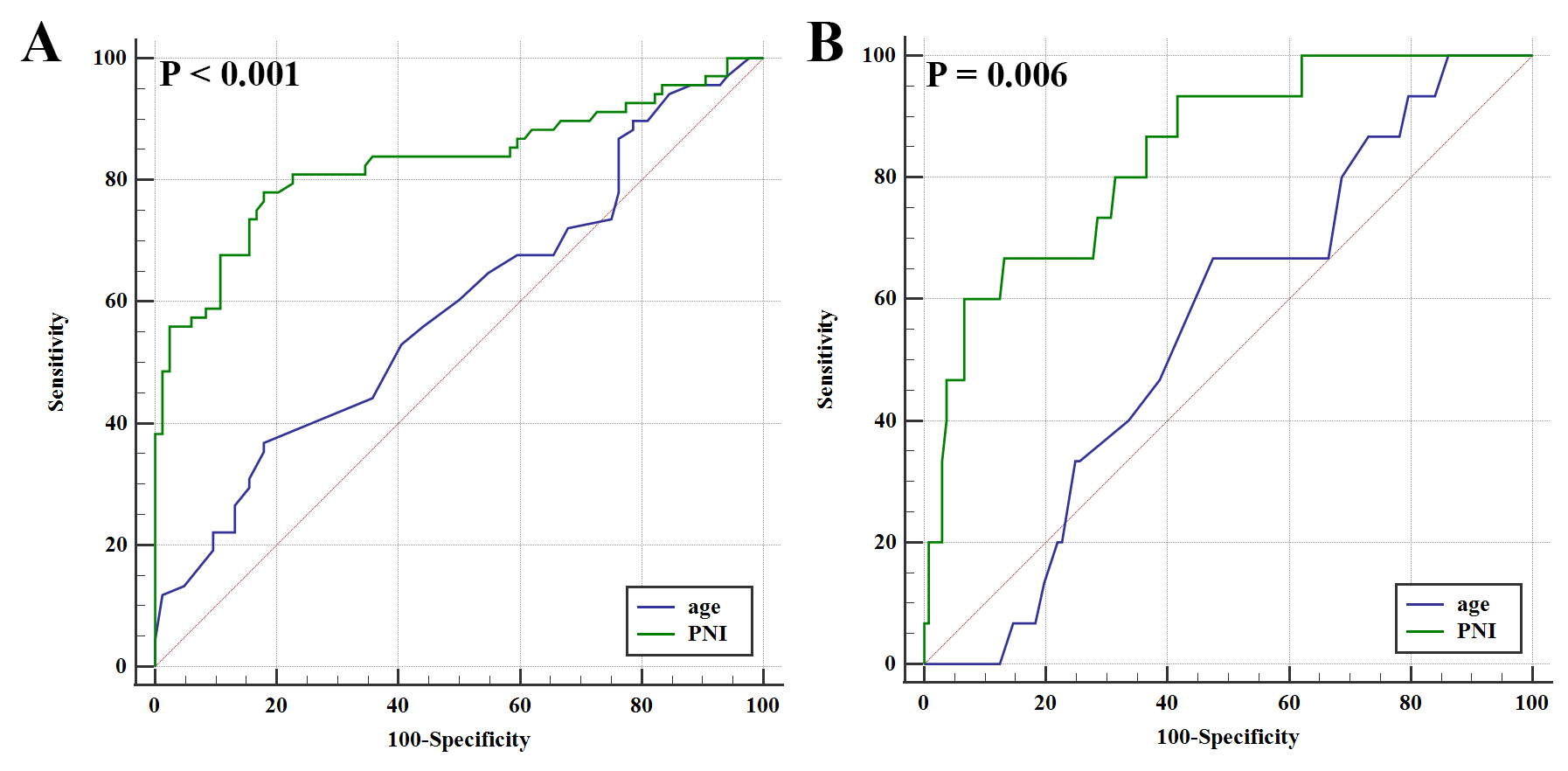

Supplement: Supplementary file 7 [file Image2.tif]
